# Viral and Atypical Bacterial Detection in Young Nepalese Children Hospitalized with Severe Pneumonia

**DOI:** 10.1128/Spectrum.00551-21

**Published:** 2021-10-27

**Authors:** Maria Mathisen, Sudha Basnet, Andreas Christensen, Arun K. Sharma, Garth Tylden, Sidsel Krokstad, Palle Valentiner-Branth, Tor A. Strand

**Affiliations:** a Department of Medical Microbiology, Drammen Hospital, Vestre Viken Hospital Trust, Drammen, Norway; b Department of Pediatrics, Institute of Medicine, Tribhuvan University, Kathmandu, Nepal; c Department of Medical Microbiology, St. Olav’s Hospital, Trondheim University Hospital, Trondheim, Norway; d Department of Clinical and Molecular Medicine, Norwegian University of Science and Technology, Trondheim, Norway; e Division of Infectious Disease Control, Norwegian Institute of Public Health, Oslo, Norway; f Department of Microbiology and Infection control, University Hospital of North Norway, Tromsø, Norway; g Statens Serum Institut, Department of Infectious Disease Epidemiology and Prevention, Infectious Disease Preparedness, Copenhagen, Denmark; h Department of Research, Innlandet Hospital Trust, Lillehammer, Norway; Children’s Hospital Los Angeles, University of Southern California

**Keywords:** respiratory viruses, atypical bacteria, lower respiratory tract infection, community-acquired pneumonia, PCR, children, low-income country, epidemiology

## Abstract

Respiratory viruses cause a substantial proportion of respiratory tract infections in children but are underrecognized as a cause of severe pneumonia hospitalization in low-income settings. We employed 22 real-time PCR assays and retrospectively reanalyzed 610 nasopharyngeal aspirate specimens from children aged 2 to 35 months with severe pneumonia (WHO definition) admitted to Kanti Childrens’ Hospital in Kathmandu, Nepal, from January 2006 through June 2008. Previously, ≥1 of 7 viruses had been detected by multiplex reverse transcription-PCR in 30% (188/627) of cases. Reanalyzing the stored specimens, we detected ≥1 pathogens, including 18 respiratory viruses and 3 atypical bacteria, in 98.7% (602/610) of cases. Rhinovirus (RV) and respiratory syncytial virus (RSV) were the most common, detected in 318 (52.1%) and 299 (49%) cases, respectively, followed by adenovirus (AdV) (10.6%), human metapneumovirus (hMPV) (9.7%), parainfluenza virus type 3 (8.4%), and enterovirus (7.7%). The remaining pathogens were each detected in less than 5%. Mycoplasma pneumoniae was most common among the atypical bacteria (3.7%). Codetections were observed in 53.3% of cases. Single-virus detection was more common for hMPV (46%) and RSV (41%) than for RV (22%) and AdV (6%). The mean cycle threshold value for detection of each pathogen tended to be lower in single-pathogen detections than in codetections. This finding was significant for RSV, RV, and AdV. RSV outbreaks occurred at the end of the monsoon or during winter. An expanded diagnostic PCR panel substantially increased the detection of respiratory viruses in young Nepalese children hospitalized with severe pneumonia.

**IMPORTANCE** Respiratory viruses are an important cause of respiratory tract infections in children but are underrecognized as a cause of pneumonia hospitalization in low-income settings. Previously, we detected at least one of seven respiratory viruses by PCR in 30% of young Nepalese children hospitalized with severe pneumonia over a period of 36 months. Using updated PCR assays detecting 21 different viruses and atypical bacteria, we reanalyzed 610 stored upper-respiratory specimens from these children. Respiratory viruses were detected in nearly all children hospitalized for pneumonia. RSV and rhinovirus were the predominant pathogens detected. Detection of two or more pathogens was observed in more than 50% of the pneumonia cases. Single-virus detection was more common for human metapneumovirus and RSV than for rhinovirus and adenovirus. The concentration of virus was higher (low cycle threshold [*C_T_*] value) for single detected pathogens, hinting at a high viral load as a marker of clinical significance.

## INTRODUCTION

After the neonatal period, pneumonia remains the leading cause of death among children less than 5 years old, with an estimated 0.9 million deaths globally in 2015. Most of these deaths occur in developing countries (1). Knowledge about the etiology of pneumonia is therefore crucial for planning future preventive and treatment measures. While a wide range of different bacteria and viruses are associated with childhood pneumonia (2, 3), clinically, these entities are mostly indistinguishable. Bacterial causes, especially Streptococcus pneumoniae and, to a lesser degree, Haemophilus influenzae, have traditionally been regarded as the most important (4), but the true role of bacteria in pneumonia is difficult to assess due to the lack of standardized diagnostic tools (5). Moreover, the widespread implementation of routine vaccination against Haemophilus influenzae type b (Hib) and S. pneumoniae has likely altered the distribution of pathogens in severe childhood pneumonia (6, 7).

Respiratory viruses cause the majority of acute lower respiratory infection (ALRI), including pneumonia, in children, and this is especially pronounced for those under the age of 5 (8–15). And yet, until recently, no pathogens could be identified in many suspected infections. Earlier virological diagnostic techniques lacked sensitivity and led to an underestimation of the role of viruses in pneumonia (16–19). Some respiratory viruses do not grow in culture and can only be readily detected by nucleic acid-based techniques, including PCR. Advances in molecular diagnostics have increased our ability to detect and characterize respiratory viruses, enhancing our understanding of their epidemiology. Rhinovirus (RV) and coronavirus (CoV), previously considered causes of the common cold, have increasingly become recognized as agents of ALRI (20, 21). Molecular methods have also aided in the discovery of several new respiratory viruses during the past 2 decades, such as human metapneumovirus (hMPV), human bocavirus 1 (BoV), the pandemic influenza A virus (IA) (H1N1)pdm09, and several CoVs, i.e., severe acute respiratory syndrome coronavirus 1 (SARS-CoV-1), Middle East respiratory syndrome coronavirus (MERS-CoV), CoV-NL63, CoV-HKU1, and finally, SARS-CoV-2.

The development of multiplex real-time PCR assays has enabled the simultaneous detection of a wide range of viral and atypical bacterial pathogens (18, 22, 23). The Hexaplex assay (Prodesse, now GeneProbe), which detected six viruses, was the first commercially available multiplex assay to be developed (24–27). More recently, multiplex assays that detect >30 respiratory pathogens have been introduced. In addition to broadening the scope of respiratory surveillance studies, multiplex assays have revealed the simultaneous presence of multiple coinfecting pathogens in nasopharyngeal aspirate (NPA) specimens from children. Broad respiratory panels have largely become the standard for identification of respiratory pathogens in hospital settings in high-income regions (5, 19, 23). However, respiratory viruses remain underrecognized as a cause of severe pneumonia requiring hospitalization in low-income settings, where access to molecular diagnostic tests is limited.

This study describes the epidemiology of respiratory viruses and atypical bacteria in young children hospitalized with pneumonia in a low-income country over a period of 2 1/2 years. The collected NPA specimens were originally analyzed using the Hexaplex plus assay (28). In an attempt to improve on previous results, we reanalyzed 610 of the original NPA specimens, employing 22 real-time PCR assays. The assays included the seven RNA respiratory viruses detected in the previous study and an additional 14 pathogens, comprising 11 respiratory viruses and 3 atypical bacteria. We hypothesized that an expanded diagnostic panel based on modern molecular methods would improve the diagnostic yield substantially and identify potential pathogens in a larger proportion of our study participants.

## RESULTS

### Study participants.

The mean (standard deviation [SD]) age of participants was 7.4 (5.8) months; 82.5% were infants aged 2 to 11 months, with more boys (61%). Nearly all children were still breastfed. About a quarter of children were wasted (Z score of <−2 for weight-for-length/height) (Table 1). Seventy percent of participants lived within the Kathmandu Valley (28).

**TABLE 1:** Demographic, anthropometric, and clinical characteristics of cases of severe pneumonia in young children hospitalized in Nepal^*a*^

Characteristic	No. of cases with data available	Value
Demographic data		
Age in mo [mean (SD)]	610	7.4 (5.8)
No. (%) who were:		
Infants	610	503 (82.5)
Boys	610	371 (60.8)
Currently breastfed	610	582 (95.4)
Age of mother in yr [mean (SD)]	587	24.4 (4.1)

Anthropometric data [no. (%)]^*b*^		
Wasted (<−2 WHZ)	595	157 (26.4)
Stunted (<−2 HAZ)	609	50 (8.2)

Clinical data		
Duration [median (IQR)] of:		
Cough (days)	610	4 (3, 5)
Difficult breathing (h)	610	24 (15, 48)
Fever (days)	554	3 (2, 5)
General danger signs [no. (%)]^*c*^	610	
Unable to drink/breastfeed		58 (9.5)
History of convulsions		4 (0.7)
Vomiting everything he/she eats		16 (2.6)
Unconscious/lethargic		57 (9.7)
Respiratory rate in breaths/min [mean (SD)] in children aged^*d*^:		
2–11 mo	503	65 (11.7)
12–35 mo	107	61 (11.8)
Axillary temp [no. (%)] of:		
>37.5°C	610	313 (51.3)
>38.5°C	610	95 (15.6)
No. (%) with:		
Wheezing	610	502 (82.3)
Crepitations	610	557 (91.3)
Oxygen saturation of <90%^*e*^	610	381 (62.5)
Nasal flaring	609	241 (39.6)
Grunting	610	135 (22.1)
Head nodding	610	141 (23.1)
Mean hemoglobin in g/dl (SD)	610	10.7 (1.3)
C-reactive protein in mg/liter	584	
Median (IQR)		20.1 (7.1, 46.8)
No. (%) with:		
>40 mg/liter		175 (30.0)
>80 mg/liter		79 (13.5)
Radiographic consolidation [no. (%)]	459	166 (36.2)

a Severe pneumonia was diagnosed according to the WHO definition in children 2 to 35 months old admitted to Kanti Children’s Hospital, Kathmandu, Nepal, from January 2006 to July 2008. SD, standard deviation; IQR, interquartile range.

b Calculated using the WHO Child Growth Standards 2005 (72). WHZ, weight-for-length/height Z score; HAZ, length/height-for-age Z score.

c As defined by the WHO Integrated Management of Childhood Illness (68).

d The lower of two counts.

e The higher of two measurements.

### Clinical characteristics.

About half of the cases presented with fever (axillary temperature of >37.5°C), 82% had wheezing, and 91% had crepitations on auscultation. In addition to lower chest wall indrawing, half of the children also presented with additional severity signs, such as nasal flaring, grunting, or head nodding, and 19% had at least one of four danger signs (Table 1). Sixty-two percent were hypoxic (O_2_ saturation of <90%). Among the 584 who had their plasma C-reactive protein (CRP) concentration measured, the median (interquartile range [IQR]) CRP concentration was 20.1 (7.1 to 46.8) mg/liter; 175 (30%) had CRP concentrations of >40 mg/liter, and 79 (13.5%) had CRP concentrations of >80 mg/liter (Table 1). Radiographic pneumonia, defined as endpoint consolidation on chest X-ray, was detected in 166 of 459 (36.2%) chest radiographs available for interpretation. Selected clinical features by pathogen are shown in Table S1 in the supplemental material.

### Respiratory pathogen detection.

We reanalyzed 610 of the previously collected specimens, employing 22 PCR assays. The expanded panel included the seven respiratory viruses in the original study and an additional 14 pathogens. The retesting resulted in a pathogen being detected in 602 of 610 (98.7%) cases, compared to 188 of 627 (30%) cases in the on-location analyses in Nepal (Table 2).

**TABLE 2:** Respiratory pathogen detections in NPA specimens from young Nepalese children hospitalized with severe pneumonia^*a*^

Pathogen^*b*^	Previous analyses (*n* = 627)		New analyses (*n* = 610)	
	No. of detections	% (95% CI)	No. of detections^*c*^	% (95% CI)
RSV	88	14.0 (11.4–17.0)	299	49.0 (45.0–53.1)
hMPV	9	1.4 (0.70–2.7)	59	9.7 (7.4–12.3)
PIV-1	23	3.7 (2.3–5.4)	22	3.6 (2.3–5.4)
PIV-2	5	0.80 (0.26–1.9)	6	1.0 (0.36–2.1)
PIV-3	24	3.8 (2.5–5.6)	51 (*n* = 604)	8.4 (6.4–11.0)
PIV-4	NA	–	16 (*n* = 608)	2.6 (1.5–4.2)
Influenza A virus	28	4.5 (3.0–6.4)	28	4.6 (3.1–6.6)
Influenza B virus	17	2.7 (1.6–4.3)	13	2.1 (1.1–3.6)
Influenza C virus	NA		8 (*n* = 608)	1.3 (0.57–2.6)
RV	NA		318	52.1 (48.1–56.2)
AdV	NA		64 (*n* = 605)	10.6 (8.2–13.3)
CoV-OC43	NA		16	2.6 (1.5–4.2)
CoV-NL63	NA		13 (*n* = 579)	2.2 (1.2–3.8)
CoV-229E	NA		3	0.50 (0.10–1.4)
CoV-HKU1	NA		3 (*n* = 607)	0.50 (0.10–1.4)
Enterovirus	NA		47 (*n* = 608)	7.7 (5.7–10.1)
Parechovirus	NA		21 (*n* = 608)	3.5 (82.1–5.2)
Bocavirus	NA		27 (*n* = 608)	4.4 (2.9–6.4)
Mycoplasma pneumoniae	NA		22 (*n* = 602)	3.7 (2.3–5.5)
Chlamydophila pneumoniae	NA		7	1.1 (0.46–2.4)
Bordetella pertussis	NA		2	0.33 (0.04–1.1)
Any pathogen	188	30 (26.4–33.7)	602	98.7 (97.4–99.4)

a NPA specimens were collected from Nepalese children aged 2 to 35 months hospitalized with severe pneumonia according to the WHO definition. Results of molecular testing using previous laboratory assays and updated new assays are shown. NPA, nasopharyngeal aspirate; CI, confidence interval; NA, not applicable.

b RSV, respiratory syncytial virus; PIV, parainfluenza virus; hMPV, human metapneumovirus; RV, rhinovirus; AdV, adenovirus; CoV, coronavirus.

c The total number of cases for which data were available is shown if *n* < 610 due to missing data.

Analyses for RSV, hMPV, parainfluenza virus types 1 to 3 (PIV-1 to -3), and IA and influenza B virus (IB) had already been performed on this population. The updated PCR assays resulted in 284 additional detections of these seven viruses (194 versus 478), while the 14 new PCR assays yielded 611 new detections. The updated assays failed to detect one case each of PIV-3 and hMPV, two cases of PIV-1, three cases of IA, four cases of RSV, and six cases of IB.

We observed that the mean cycle threshold (*C_T_*) values were lower for the specimens that were positive both in the previous analyses and in the reanalysis compared to the mean *C_T_* values of specimens that were positive in the reanalysis only, with mean differences in *C_T_* values of 1.8 for RSV (*P* = 0.005), 3.4 for hMPV (*P* = 0.09), and 5.0 for PIV-3 (*P* = 0.003).

Rhinovirus and RSV were the most frequent pathogens in the study, detected in 318 (52.1%) and 299 (49%) cases, respectively, followed by AdV (10.6%), hMPV (9.7%), PIV-3 (8.4%), and enterovirus (7.7%). Each of the other pathogens was detected in less than 5% of cases. Among the atypical bacteria, Mycoplasma pneumoniae was most common, identified in 22 cases (3.7%).

A single pathogen was detected in 45.3% of the study specimens (*n* = 276), two pathogen in 38% (*n* = 232), three pathogen in 12% (*n* = 73), and four or more pathogen in 3.4% (*n* = 21). RSV, hMPV, IA and IB were detected as single pathogen more often than RV, PIV-3, AdV, and BoV, which were more often present in codetections (Table 3). Enterovirus, parechovirus (PeV), and CoV-NL63 were not detected alone/as single pathogen in any of the specimens. Neither were PIV-2, influenza C virus, CoV 229E, and CoV-HKU1, but they were each detected in <10 cases. Single detection was seen for CoV-OC43 in three cases. Among the atypical bacteria, only 4 of 22 specimens with M. pneumoniae were single detections, and none of the 7 C. pneumoniae or 2 B. pertussis isolates were detected alone.

**TABLE 3:** Single and codetections by pathogen in NPA specimens from young Nepalese children hospitalized with severe pneumonia^*a*^

Pathogen^*b*^	Total no. of detections	No. (%) of cases that had:			
		Single detections	Double detections	Triple detections	≥4 detections
RSV	299	122 (40.8)	129 (43.1)	36 (12.0)	12 (4.0)
hMPV	59	27 (45.8)	19 (32.2)	10 (17.0)	3 (5.1)
PIV-1	22	6 (27.3)	9 (40.9)	5 (22.7)	2 (9.1)
PIV-2	6	0	2 (33.3)	2 (33.3)	2 (33.3)
PIV-3	51	13 (25.5)	24 (47.1)	9 (17.7)	5 (9.8)
PIV-4	16	1 (6.3)	8 (50.0)	6 (37.5)	1 (6.3)
Influenza A virus	28	13 (46.4)	9 (32.1)	4 (14.3)	2 (7.1)
Influenza B virus	13	7 (53.9)	3 (23.1)	3 (23.1)	0
Influenza C virus	8	0	4 (50.0)	3 (37.5)	1 (12.5)
Rhinovirus	318	71 (22.3)	169 (53.1)	59 (18.6)	19 (6.0)
Adenovirus	64	4 (6.3)	30 (46.9)	20 (31.3)	10 (15.6)
CoV-OC43	16	3 (18.8)	9 (56.3)	3 (18.8)	1 (6.3)
CoV-NL63	13	0	6 (46.2)	4 (30.8)	3 (23.1)
CoV-229E	3	0	1	2	0
CoV-HKU1	3	0	2	1	0
Enterovirus	47	0	10 (21.3)	21 (44.7)	16 (34.0)
Parechovirus	21	0	8 (38.1)	11 (52.4)	2 (9.5)
Bocavirus	27	5 (18.5)	10 (37.0)	8 (29.6)	4 (14.8)
Mycoplasma pneumoniae	22	4 (18.2)	9 (40.9)	7 (31.8)	2 (9.1)
Chlamydophila pneumoniae	7	0	3 (42.9)	3 (42.9)	1 (14.3)
Bordetella pertussis	2	0	0	2	0

a NPA specimens were collected from 610 Nepalese children aged 2 to 35 months hospitalized with severe pneumonia according to the WHO definition. NPA, nasopharyngeal aspirate.

b RSV, respiratory syncytial virus; PIV, parainfluenza virus; hMPV, human metapneumovirus; CoV, coronavirus.

For RSV, hMPV, IA, RV, AdV, and BoV, the mean *C_T_* value tended to be lower in single-pathogen detection than in codetection. This finding was significant for RSV, RV, and AdV (Table 4). The mean *C_T_* value for RSV was lower than that of RV both in the single-detection group and in the codetection group. Moreover, the mean difference in *C_T_* values between single detections and codetections was three times greater for RV than for RSV, 4.1 versus 1.3, respectively, reflecting a greater variability of RV *C_T_* values overall. In addition, the mean *C_T_* value of RV was higher when codetected with RSV than when not (31.8 versus 30.0).

**TABLE 4:** Difference in PCR cycle threshold values for single detections versus codetections in selected viruses from young Nepalese children hospitalized with severe pneumonia^*a*^

Virus^*b*^	Total no. of detections	Single detections		Codetections		Difference in mean *C_T_* values (95% CI)	*P* value
		No.	Mean *C_T_* value (SD)	No.	Mean *C_T_* value (SD)		
RSV	299	122	22.9 (2.9)	177	24.2 (4.6)	1.3 (0.40–2.3)	0.008
hMPV	59	27	27.1 (3.7)	32	27.9 (4.6)	0.8 (−1.4–3.0)	0.465
PIV-3	51	13	27.2 (6.3)	38	27.0 (5.5)	−0.25 (−3.9–3.4)	0.891
Influenza A virus	28	13	29.0 (2.1)	15	31.0 (3.8)	2.1 (−0.37–4.5)	0.092
RV	318	71	26.9 (5.2)	247	30.9 (4.5)	4.1 (2.8–5.4)	<0.0001
Adenovirus	64	4	23.6 (4.8)	60	33.1 (6.3)	9.5 (3.0–15.9)	0.005
Bocavirus	27	5	19.4 (7.3)	22	24.8 (7.1)	5.4 (−3.5–14.3)	0.138

a PCR cycle threshold values were compared for single detections versus codetections in selected viruses detected in NPA specimens from 610 Nepalese children aged 2 to 35 months hospitalized with severe pneumonia according to the WHO definition. Data are for viruses with >25 detections. NPA, nasopharyngeal aspirate; *C_T_* value, cycle threshold value.

b RSV, respiratory syncytial virus; PIV, parainfluenza virus; hMPV, human metapneumovirus; RV, rhinovirus.

### Viral seasonal distribution.

**FIG 1:**
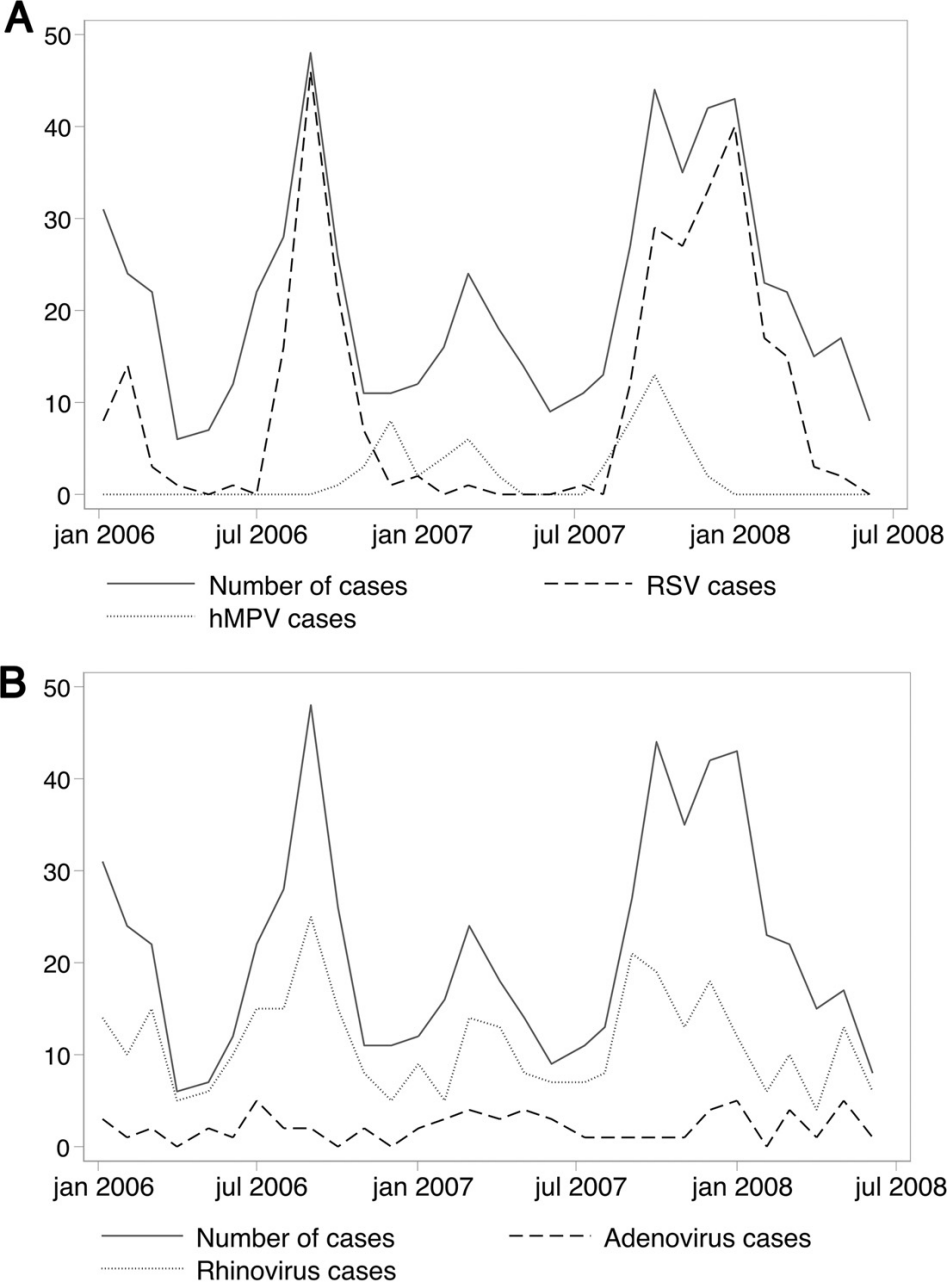
Monthly numbers of cases with community-acquired severe pneumonia (WHO) and cases testing positive for RSV and hMPV (A) or RV and AdV (B) among 610 children 2 to 35 months old admitted to Kanti Children’s Hospital, Kathmandu, Nepal, from January 2006 to June 2008.

During the 30-month study period, RSV epidemics occurred at the end of the monsoon (September to October) or during winter (November to February). The first RSV outbreak peaked in September (2006) and was over by December, while the following year, there was a smaller peak in October but the outbreak was prolonged; it reached its maximum peak in January and did not end until April (2007) (Fig. 1A). We also observed RSV activity at the beginning of the study that peaked in February, possibly representing the tail end of a larger epidemic starting before the study period.

The hMPV activity occurred in epidemics similar to those of RSV but with lower magnitudes. The first hMPV outbreak lasted from October 2006 through April 2007, with two peaks in December and March. The second outbreak occurred from August to December 2007 with a peak in October. Neither RSV nor hMPV was detected in significant numbers between epidemics, in contrast to RV and AdV, which were detected throughout the study, AdV evenly throughout the year and RV with various epidemic peaks (Fig. 1B). RV outbreaks peaked in autumn (September in both years), winter (December 2007), and spring (March 2006), and the two major RV peaks coincided with or substantially overlapped the RSV outbreaks.

## DISCUSSION

Retrospective reanalysis of previously collected NPA specimens with an expanded real-time PCR panel including 21 respiratory pathogens resulted in 895 additional detections, of which 284 were new detections of the previously analyzed viruses and 611 were detections of the 14 newly added microbial targets.

Retesting for the original seven viruses with updated assays increased the number of detections substantially, from 194 in 627 cases to 478 in 610 cases. Using real-time multiplex PCR methods, the detections of hMPV increased more than six times, detections of RSV increased three times, and detections of PIV-3 increased two times, whereas IA, IB, PIV-1, and PIV-2 were detected in similar numbers as in the previous analyses, suggesting that the Hexaplex plus assay lacked sensitivity, especially with regard to RSV, hMPV, and PIV-3. Specimens that tested positive in reanalysis only had higher mean *C_T_* values, supporting this notion. However, detailed calculation of the limits of detection (LODs) for the qualitative Hexaplex plus assays and semiquantitative real-time assays was not performed at the time of local validation. Direct comparison of the LODs of the old and new methods was not possible, as the Hexaplex plus assay was no longer in use in the routine diagnostic laboratory at the time of reanalysis.

After reanalysis with the expanded respiratory panel, we detected respiratory viruses in 98% of pneumonia cases. This detection rate is higher than those reported by two comparable studies (29, 30). These comprehensive multicenter pneumonia etiology studies in low- and middle-income countries (LMICs) reported detection of one or more viruses by PCR in specimens from the upper respiratory tract in 89% and 78% of hospitalized children <5 years of age with severe pneumonia. Both studies used multiplex real-time PCR and tested for a range of respiratory viruses similar to that in the current study.

The higher proportion of viral detections in the current study was mainly due to RSV and RV, which were the most frequently detected by far, both present in about half of the cases. This is a larger proportion than in the two above-mentioned studies, which detected RSV in 20 to 26.5% and RV in 23 to 25% of pneumonia cases (29, 30). These discrepancies may be explained by differences in age distribution, case definition, study timing, and test sensitivity. Notably, both studies demonstrated substantial variation in the detection of pathogens between countries.

The incidence of viral infection in children hospitalized with community-acquired pneumonia (CAP) is significantly higher in those <2 years of age than in those between 2 and 4 years of age (10, 31, 32). This is even more pronounced in the first year of life, when RSV plays a pivotal role (29, 30). Our study population consisted of children <3 years old, with 82.5% aged 2 to 11 months, which is a higher proportion than in the two LMIC studies cited above.

The pneumonia case definition (WHO Integrated Management of Childhood Illnesses [IMCI] guidelines) used in this study was designed for high sensitivity in a low-resource setting and will consequently identify most episodes of ALRI, including bronchiolitis and pneumonia (33). RSV is known to be the most common cause of acute bronchiolitis in young children, followed by RV, and some studies of acute bronchiolitis have detected RSV in up to 70 to 80% of cases and RV in up to 34% (34–37).

The magnitudes of RSV and RV outbreaks depend on a complex interplay between susceptibility in the population, climatic factors, and circulating viral subtypes, among other factors (38–41), and may vary widely between studies.

In this study, we emphasized optimal sensitivity for RV detection, including all the newly discovered rhinovirus species C (RV-C) strains. This increased the RV test sensitivity considerably.

The proportions of other pathogens detected in the current study were in line with the other two studies (29, 30), except that we detected bocavirus less frequently (4.4% versus 9.2% and 13.1%) and M. pneumoniae somewhat more frequently (3.7% versus 1.1% and 1.5%.).

Codetections occurred in 53.3% of cases in the current study. Multiple detections are common in respiratory specimens of young children with acute respiratory infection (ARI). In epidemiological studies based on molecular detection methods, at least two viruses or atypical bacteria have been reported in 12 to 40% of cases (10, 12, 15, 42–44). Codetections vary across age groups and are more common in younger children, who usually are more susceptible to viral infections and have higher viral loads in their respiratory secretions (45, 46). Moreover, RVs are shed for longer time periods; after the onset of symptomatic respiratory infection, rhinovirus RNA may take up to 5 to 6 weeks to disappear from the nasal secretions of children (47). Seasonal circulation patterns will also influence codetections, as well as overall detections, explaining the differences between studies.

We observed 133 codetections of RSV and RV. RV demonstrated an endemic seasonal pattern with detections throughout the year, and RV peaks coincided/overlapped with RSV outbreaks (Fig. 1). One could speculate that RV might be a “bystander” in some children with RSV, as RSV detections during RSV outbreaks would be the most likely cause for the hospital admission (48). In addition, RSV was more often detected as a single infection than was RV, and the mean *C_T_* value for RSV was significantly lower than that of RV in codetections.

Respiratory syncytial virus, hMPV, IA, and IB were detected as single pathogen more often than RV, PIV-3, AdV, CoV, and BoV, which were frequently identified in codetections. Some viruses were not detected alone in any of the specimens, including enterovirus (EV), PeV, and CoV-NL63. It has been shown that AdV, RV, EV, and BoV can be detected for longer periods in the airways, due to asymptomatic carriage, persistent shedding after infection (47, 49, 50), or reactivation of latent infection (51, 52), which may explain why these viruses are more often found in codetections. Unexpectedly, PIV-3, a virus well known to cause ALRI in young children (53), was also more frequently found in combination with other pathogens. Studies of CoV in children with ARI have reported that nearly half to two-thirds of CoV detections were codetections, including in Nepalese children (21, 54). Similar findings have been reported for AdV in Norwegian children with ARI (55).

Adenovirus, BoV, and CoV are frequently found in combination with other viruses and in healthy controls (21, 55–57), making clinical evaluation difficult. Qualitative PCR has limited value in the diagnosis of such infections. Quantitative PCR, mRNA detection, or supplemental serology may help establish causality (21, 55, 57–59).

The frequency of codetections and the fact that many of the pathogens in question are also commonly detected in asymptomatic children highlights the need for a case-control design in epidemiological studies to quantify the etiological contributions of individual pathogens in pneumonia (60). A review and meta-analysis of 23 case-control studies published from 1990 to 2014 estimated that there was strong evidence of causality for RSV and less strong evidence for RV in children <5 years with ALRI (48). This was confirmed in the two more recent case-control studies cited above (29, 30). The meta-analysis also found strong evidence of a causal role for influenza virus, hMPV, and PIV, whereas no difference between cases and controls was seen for the detection of AdV, BoV, or CoV (48). This is mainly in line with the tendency seen in our findings regarding single and codetected pathogens.

M. pneumoniae was detected alone in only 4 of the 22 cases that tested positive for this pathogen. Others have reported a relative infrequency of codetections between M. pneumoniae and respiratory viruses (32). Using the Biofire FilmArray, Zheng and coworkers reported that 26/80 (33%) respiratory specimens from symptomatic children had a viral pathogen detected along with M. pneumoniae (61). Using a case-control design, Spuesens et al. demonstrated that M. pneumoniae was present in the upper respiratory tract of 21% of asymptomatic children aged 3 months to 16 years (62), suggesting that asymptomatic carriage of this atypical bacterium is not uncommon and could explain its involvement in codetections.

### Seasonality.

We have proposed previously that RSV activity follows a biennial rhythm in the Kathmandu Valley, with alternating early and late onset of epidemics, similar to what has been observed in Europe (28). In southern Nepal, Perchetti et al. reported hMPV infections to occur during fall and winter months with detections starting in September or October and in two of three seasons extending into March/April (63), similar to our findings. The larger RV outbreaks peaked in September both years, and a smaller peak was seen during winter or spring. Others have previously described RV infections appearing year-round with peaks in early autumn and spring (38, 64, 65), a pattern that has been associated with the circulation of different RV types, creating simultaneous or successive epidemics (66).

### Limitations.

The main limitation of our study is that it was embedded in a clinical trial of zinc as an adjunct treatment for severe pneumonia (67) and, therefore, was subject to the eligibility criteria of the main trial. The major consequence of this was that not all children with severe pneumonia admitted to the Kanti Children’s Hospital were included during the study period, consequently making our findings somewhat less generalizable. A history of recurrent wheezing, nonconsent, heart disease, or other severe conditions were the main reasons for exclusion (67).

Pneumococcal conjugate vaccine (PCV) has been reported to reduce the risk of virus-associated pneumonia (6). Vaccines against Hib and S. pneumoniae were introduced in the routine infant immunization program in Nepal in 2009 and 2015, respectively, i.e., after the collection of specimens for this study. The implementation of these vaccines may have altered the local distribution of pathogens in severe childhood pneumonia.

The enrollment of the study participants was conducted over 30 months. Despite being longer than most studies, it may still be too short to capture pathogens whose epidemiology is greater than two-and-a-half-year cycles.

### Conclusion.

In summary, using a broad respiratory PCR panel, we were able to detect a respiratory pathogen in almost all children 2 to 35 months old with severe pneumonia. The study was conducted in a large, well-defined population of young, hospitalized children in a subtropical-to-temperate low-income country. Further studies, preferably with a case-control design and quantitative detection methods, are warranted to elucidate the local epidemiology of respiratory viruses in the era of routine use of PCV and the Hib vaccine to inform future strategies for prevention and treatment of pneumonia in low-income settings.

## MATERIALS AND METHODS

### Study subjects, case definition, and specimen collection and handling.

The original study was conducted between 1 January 2006 and 30 June 2008 at Kanti Children’s Hospital in Kathmandu, a tertiary referral hospital for the whole of Nepal. Children aged 2 to 35 months presenting to the hospital were screened for eligibility. Severe pneumonia was defined as cough or difficult breathing combined with lower chest wall indrawing (LCI), according to the Integrated Management of Childhood Illnesses (IMCI) algorithm for acute respiratory infections (68). Moreover, children with wheezing were given up to 3 doses of nebulized salbutamol 15 min apart and were not found eligible if LCI disappeared on reassessment (69). Additional details of participant selection and other study procedures can be accessed in a previous publication (28).

Study physicians collected NPA specimens from all children upon enrollment in the study. Immediately following collection, the NPA specimen was divided into three aliquots and stored at −70°C. While one aliquot was analyzed on location in Nepal, the remaining two aliquots were later shipped to Norway on dry ice.

### Previous PCR methods and results.

A total of 627 children with severe pneumonia included in the original study from whom NPA specimens were collected at presentation had a valid PCR result (70). Each specimen was tested for RSV, influenza A and B viruses, PIV-1, -2, and -3, and hMPV using a commercially available multiplex reverse transcription-PCR assay (Hexaplex Plus, Prodesse, Inc., Waukesha, WI). We identified ≥1 respiratory virus in 188 of the 627 cases (30%), whereas in 439 cases (70%), no virus was detected (28). Of the 627 previously collected NPA specimens stored at −70°C, 610 were available for reanalysis.

### PCR methods for the reanalysis.

At the Department of Microbiology and Infection Control, University Hospital of North Norway, nucleic acids were extracted automatically (NucliSens easyMAG [bioMérieux] or Chemagic STAR [Hamilton]). Simultaneous triplex or duplex real-time reverse transcription-PCR analyses were performed for PIV-1, -2, and -3, influenza B virus and respiratory syncytial virus, human metapneumovirus and human coronavirus NL63, influenza A virus and human coronavirus OC43, and rhinovirus and human coronavirus 229E using previously described primers, probes, and cycling conditions (22). In-house real-time PCR analyses were performed for adenovirus, Mycoplasma pneumoniae, Chlamydia pneumoniae, and Bordetella pertussis using TaqMan probes (Roche Diagnostics, Basel, Switzerland). All amplifications were carried out in the ABI 7500 real-time PCR system (Applied Biosystems). Analyses were performed during 2014.

To test for additional viruses, nucleic acids were extracted automatically (MagNA Pure 96; Roche Diagnostics, Basel, Switzerland) at the Department of Microbiology, Lillehammer Hospital. All 610 lysates were tested with PCRs for additional viruses, *viz.,* human coronavirus HKU1, human bocavirus 1, enterovirus, parechovirus, influenza C virus, PIV-4, and rhinovirus species A to C at the Department of Medical Microbiology, St. Olav’s Hospital, Trondheim, Norway. The PCRs were in-house, TaqMan real-time assays (Table 5) (71). Amplifications were performed on the CFX96 real-time system (Bio-Rad). Analyses were performed during 2017 to 2018.

**TABLE 5:** Primer and probe sequences for rhinovirus species A to C PCRs performed at St. Olav’s Hospital, Trondheim, Norway

Name	Sequence
HRV_A-C sense	CAGGGTGTGAAGACHCKAGTGTG
HRV_A-C as	AACACGGACACCCAAAGTAG
HRV_A-C_TM	CCCCTGAATGYGGCTAACCTTAAYC

The PCR results were considered positive for all sigmoidal curves and cycle thresholds (*C_T_*) of <40 cycles. All positive RV-A to -C results were evaluated for cross-reactivity due to EV infections by use of parallel EV PCR testing. Samples with weak amplification curves for RV and a positive EV PCR were regarded as RV negative.

### Statistical methods.

We analyzed the data using Stata/MP 12.0 for Macintosh (Stata Corporation, College Station, TX). The 95% confidence intervals for proportions were calculated using the “ci” command. To compare the mean *C_T_* values in single versus codetections for each pathogen, we used the two-sample *t* test. Statistical significance was defined as a *P* value of <0.05.

### Ethical issues.

The study and biobank had ethical clearance from the Nepal Health Research Council, Kathmandu, and the Western Regional Committee for Medical and Health Research Ethics, Norway (REC West 129.03). The implementation of the project was in agreement with international ethical principles for medical research involving human subjects as stated in the latest version of the Helsinki Declaration.
