# Supplementary material for: Viral and Atypical Bacterial Detection in Young Nepalese Children Hospitalized with Severe Pneumonia
Source: Microbiol Spectr. 2021 Oct 27;9(2):e00551-21. doi: 10.1128/Spectrum.00551-21 (PMC8549725; doi:10.1128/Spectrum.00551-21)
Supplement: SUPPLEMENTAL FILE 1 — Supplemental material. Download SPECTRUM00551-21_Supp_1_seq3.pdf, PDF file, 0.3 MB [file spectrum00551-21_supp_1_seq3.pdf]

Table 1.Selected clinical features by pathogen detected in NPA from 610 Nepalese children aged 2-35 months with severe pneumonia (WHO)

Pathogen	n/N[a]	mean age (sd) N=610	fever[b] (%) N=610	SpO2 <90% (%) N=610	n/N[c]	mean CRP (sd) N=584	n/N[d]	consolidation (%) N=459
RSV	299	6.2 (4.7)	42 (14.1)	209 (69.9)	286	29.1 (40.2)	227	88 (38.8)
hMPV	59	7.8 (5.7)	12 (20.3)	38 (64.4)	51	41.3 (49.2)	47	19 (38.3)
PIV 1	22	8.1 (6.1)	5 (22.7)	16 (72.7)	22	55.8 (47.3)	15	7 (46.7)
PIV 2	6	13.8 (12.6)	0	5 (83.3)	6	41.3 (33.0)	3	3 (100.0)
PIV 3	51/604	9.4 (7.4)	8 (15.7)	29 (56.9)	49/580	56.9 (59.9)	43/455	15 (34.9)
PIV 4	16/608	9.7 (4.5)	5 (31.3)	9 (56.3)	16/582	42.4 (73.5)	12	1 (8.3)
Influenza A	28	8.8 (5.9)	9 (32.1)	19 (67.9)	27	72.3 (80.4)	20	10 (50.0)
Influenza B	13	9.5 (4.7)	3 (23.1)	7 (53.9)	13	60.0 (66.0)	12	8 (66.7)
Influenza C	8/608	11.4 (8.3)	2 (22.2)	5 (62.5)	8/582	35.9 (35.4)	8	2 (25.0)
RV	318	7.4 (6.2)	42 (13.2)	176 (55.4)	309	36.0 (50.1)	237	83 (35.0)
AdV	64/605	9.5 (6.1)	13 (20.3)	49 (76.5)	64/580	46.8 (66.9)	37/455	10 (27.0)
CoV OC43	16	6.6 (5.0)	5 (21.3)	13 (81.3)	16	22.0 (24.2)	14	7 (50.0)
CoV NL63	13/579	6.3 (6.8)	3 (23.1)	9 (69.2)	11/553	29.9 (18.9)	10/433	6 (60.0)
CoV 229E	3	9.3 (11.8)	0	1 (33.3)	3	42.7 (54.5)	2	0
CoV HKU1	3/607	10.3 (0.6)	2 (66.7)	2 (66.7)	3/581	165.6 (25.9)	3	3 (100.0)
Enterovirus	47/608	11.1 (6.9)	8 (17.0)	34 (72.3)	46/582	46.1 (66.7)	32	11 (34.4)
Parechovirus	21/608	7.1 (3.0)	4 (18.2)	12 (57.1)	21/582	39.1 (53.7)	17	9 (52.9)
Bocavirus	27/608	10.2 (4.9)	3 (11.1)	13 (48.2)	24/582	32.8 (38.6)	22	7 (31.8)
Mycoplasma pneumoniae	22/602	6.2 (3.3)	1 (4.6)	13 (59.1)	21/576	31.9 (37.0)	13/452	5 (38.5)
Chlamydia pneumoniae	7	7.6 (3.7)	2 (28.6)	6 (85.7)	7	64.5 (49.0)	6	4 (66.7)
Bordetella pertussis	2	2.5 (0.7)	0	0	2	48.9 (68.2)	1	1 (100.0)
Any pathogen	602	7.4 (5.8)	94 (15.6)	378 (62.8)	576	38.4 (50.9)	453	165 (36.4)
CRP > 40 mg/L	175/584	8.5 (6.0)	39 (22.3)	108 (61.7)	-	- -	131/439	65 (49.6)
CRP > 80 mg/L	79/584	9.2 (6.1)	22 (27.9)	45 (57.0)	-	- -	58/439	34 (58.6)
No pathogen	8	7.6 (6.9)	1	3	5	54.6 (101.2)	4	1

RSV, respiratory syncytial virus; PIV, parainfluenza virus; hMPV, human metapneumovirus; RV, rhinovirus; AdV, adenovirus; CoV, coronavirus; CRP,C-reactive protein

[a] If N < 610

[b] temperature ≥ 38.5°C

[c] If N < 584

[d] If N < 459
